# Expression and processing of *Plasmodium berghei* SERA3 during liver stages

**DOI:** 10.1111/j.1462-5822.2008.01162.x

**Published:** 2008-08

**Authors:** Anja Schmidt-Christensen, Angelika Sturm, Sebastian Horstmann, Volker T Heussler

**Affiliations:** Bernhard Nocht Institute for Tropical MedicineBernhard-Nocht-Str 74, 20359 Hamburg, Germany

## Abstract

Cysteine proteases mediate liberation of *Plasmodium berghei* merozoites from infected hepatocytes. In an attempt to identify the responsible parasite proteases, we screened the genome of *P. berghei* for cysteine protease-encoding genes. RT-PCR analyses revealed that transcription of four out of five *P. berghei* serine repeat antigen (PbSERA) genes was strongly upregulated in late liver stages briefly before the parasitophorous vacuole membrane ruptured to release merozoites into the host cell cytoplasm, suggesting a role of PbSERA proteases in these processes. In order to characterize PbSERA3 processing, we raised an antiserum against a non-conserved region of the protein and generated a transgenic *P. berghei* strain expressing a TAP-tagged PbSERA3 under the control of the endogenous promoter. Immunofluorescence assays revealed that PbSERA3 leaks into the host cell cytoplasm during merozoite development, where it might contribute to host cell death or activate host cell proteases that execute cell death. Importantly, processed PbSERA3 has been detected by Western blot analysis in cell extracts of schizont-infected cells and merozoite-infected detached hepatic cells.

## Introduction

Despite 100 years of malaria research, malaria remains one of the world most deadly diseases, causing more than one million deaths per year. The causative agent of malaria, the *Plasmodium* parasite, is transmitted by the bite of an infected *Anopheles* mosquito. Research on the rodent malaria parasite *P. berghei* revealed that transmitted sporozoites are deposited under the skin from where they enter blood vessels to eventually reach the liver with the blood stream (Amino *et al*., 2005). By traversing Kupffer cells (Baer *et al*., 2007a), sporozoites gain access to the liver tissue where they transmigrate through a number of hepatocytes (Mota *et al*., 2001), before finally invading one by formation of a parasitophorous vacuole (PV). Inside this vacuole, the parasite develops into a large, multinucleated body called the liver schizont. The liver schizont differentiates into several thousand merozoites that must enter blood vessels to infect red blood cells (RBCs). During this process, the host cell undergoes a number of dramatic morphological changes, observed as detachment from the neighbouring cells and eventually formation of merosomes (Sturm *et al*., 2006; Sturm and Heussler, 2007). Merosomes are merozoite-filled, host cell-derived vesicles that migrate into the adjacent blood vessel of the liver tissue and therefore seem to function as a safe transport system that delivers parasites, undetected by the immune system, into the blood stream. *In vitro, P. berghei*-infected HepG2 cells detach at the end of the exoerythrocytic stage and float into the culture media, where they also form merosomes (Sturm *et al*., 2006). This entire process can be inhibited by treating infected cells with the general cysteine protease inhibitor E64. Previously, it has also been shown that E64 blocks merozoite release from infected RBCs (Salmon *et al*., 2001; Wickham *et al*., 2003), suggesting a crucial role of cysteine protease during parasite liberation. *P. falciparum* SERA proteins are a family of nine conserved putative cysteine proteases thought to play a key role in liberation of blood stage parasites from RBCs and sporozoites from oocysts (Hodder *et al*., 2003; Bourgon *et al*., 2004; Arisue *et al*., 2007). Each examined *Plasmodium* species possesses SERAs of two major groups, specified as ‘cysteine-type SERAs’ and ‘serine-type SERAs’. Serine-type SERAs contain an active site serine residue instead of the canonical cysteine residue (Hodder *et al*., 2003; Bourgon *et al*., 2004). *P. falciparum* SERA5 (PfSERA5), also known as SERP and p126 (Delplace *et al*., 1987; Bzik *et al*., 1988; Knapp *et al*., 1989), is the best-characterized SERA protease. In a series of elegant studies it has been shown that p126 or SERA5 becomes proteolytically processed in several steps to final peptide of 50 kDa (Delplace *et al*., 1985; 1987; 1988; Debrabant and Delplace, 1989; Debrabant *et al*., 1992). Leupeptin-dependent inhibition of SERA5 processing from a 56 kDa fragment to the mature 50 kDa peptide still allowed the release of blood stage merozoites from the PVM but blocked the liberaton of merozoites from the infected cell (Debrabant and Delplace, 1989), which is a striking similarity to the inhibition of merozoite release in E64-treated infected hepatocytes (Sturm *et al*., 2006).

As the genomes of different *Plasmodium* species contain different numbers of SERA genes (Arisue *et al*., 2007; McCoubrie *et al*., 2007), there is some confusion about the nomenclature of the SERA family. We decided to adopt the names recently suggested (Arisue *et al*., 2007), although they do not match the results of homology and synteny searches (McCoubrie *et al*., 2007). For example, *P. falciparum* SERA3 is a serine-type SERA, whereas *P. berghei* SERA3 is a cysteine-type SERA.

It has been shown that immune responses against the N-terminal part of PfSERA5 correlate with immunity against malaria (Okech *et al*., 2001; Okech *et al*., 2006) and thus SERA proteases might be interesting candidates for vaccine design. Here, we describe the expression and localization of *P. berghei* cysteine-type SERA3 during late liver stages of the parasite and provide evidence for its processing.

## Results

### Cysteine proteases are involved in merozoite release from the PV in *P. berghei*-infected HepG2 cells

**Fig. 1:**
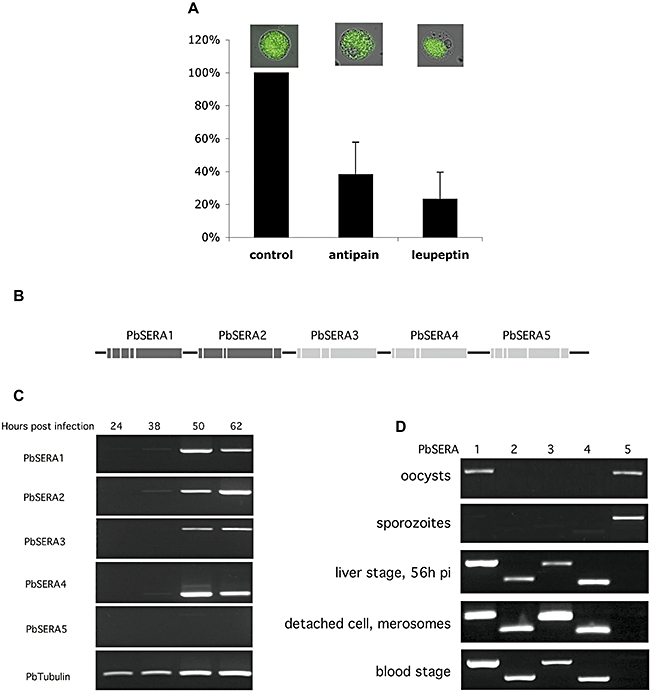
A. Cysteine and serine protease inhibitors block detachment of *P. berghei*-infected HepG2 cells. HepG2 cells were infected with *P. berghei* sporozoites. Upon cultivation for 48 h, cells were treated with 100 μM of either antipain or leupeptin for an additional 13 h. Detached cells in treated and untreated control wells were counted and expressed as a percentage of control (untreated) cells. Error bars have been calculated from data of three independent experiments. B. The SERA gene family of *P. berghei* consists of five members, aligned in a tandem cluster. Dark grey boxes represent exons of the ‘cysteine-type’ SERA members, light grey boxes symbolize the exons of ‘serine-type’ SERAs. C. PbSERA1–4 expression is upregulated in late liver stages. HepG2 cells were infected with *P. berghei* sporozoites and mRNA of these infected cells was prepared at 24, 38, 50 and 62 h post infection. Messenger RNA expression of all five PbSERAs was verified by RT-PCR. As a control, tubulin mRNA was additionally amplified. D. PbSERA mRNA expression is stage specifically regulated. mRNA expression of all five PbSERAs was verified by RT-PCR. Total mRNA was isolated from two mosquito stages (oocysts and salivary gland sporozoites), and from three parasite stages of the vertebrate host (late liver schizont stage, detached cells/merosomes and the blood stages) and subjected to RT-PCR analysis.

It has been shown for both, *Plasmodium* blood stages (Salmon *et al*., 2001; Li *et al*., 2002; Wickham *et al*., 2003) and *Plasmodium* liver stages (Sturm *et al*., 2006) that cysteine protease inhibitors can block the process of merozoite release. The liberation of exoerythrocytic *P. berghei* merozoites from the PV was effectively blocked by the irreversible cysteine protease inhibitor E64. To further characterize protease function during this process, we treated *P. berghei*-infected HepG2 cells 48 h post infection with 100 μm ml^−1^ leupeptin and antipain, two serine and cysteine protease inhibitors (Fig. 1A) and counted the detached cells 67 h after infection. Both inhibitors blocked cell detachment considerably but not completely compared with untreated cells. Infected cells that still detached despite inhibitor treatment exhibited a different phenotype depending on the drug used (inserted images in Fig. 1A). In leupeptin-treated cultures, merozoites in detached cells were still surrounded by a PVM within the host cell. On the other hand, detached cells in antipain-treated cultures looked similar to detached cells of untreated control cultures, with merozoites mixing freely with the host cell cytoplasm. These results suggest that the inhibitors either act on different proteases or inhibit the same protease by different mechanisms. It has indeed been shown that leupeptin and antipain have different inhibitory effects on defined proteases (Otto and Schirmeister, 1997). The fact that leupeptin treatment preserved the PVM structure in infected detached cells whereas in antipain-treated detached cells merozoites were liberated from the PV supports the hypothesis that more than one protease is involved in parasite liberation and induction of host cell death. One likely scenario is that a kind of ‘initiator’ protease induces the processing of ‘effector’ proteases and their activation results in the observed phenotype. Depending on which stage the individual inhibitors act, destruction of the PVM, cell detachment or host cell death might be affected.

### Transcription of four SERA genes is strongly upregulated in late liver stages

We reasoned that proteases that are responsible for PVM destruction and merozoite liberation into the host cell cytoplasm are most likely of parasite origin and thus, we performed database searches for *Plasmodium* cysteine proteases containing a signal sequence. To focus on candidate proteases relevant for merozoite liberation, a second selection criterion was the proof of an upregulated transcription towards the end of the liver stage, briefly before PVM damage. Reverse transcription polymerase chain reaction (RT-PCR) analysis revealed a strongly enhanced expression of five cysteine protease mRNAs in late liver stages, including a putative cysteine protease annotated as PB000888.02.0 (data not shown) and four PbSERA proteases (Fig. 1C). Unlike *P. falciparum*, which possesses nine SERA protease genes (Miller *et al*., 2002), the *P. berghei* genome contains only five (Fig. 1B and Fig. S1) (Bourgon *et al*., 2004; Arisue *et al*., 2007), with PbSERA1–4 but not PbSERA5 expressed in late liver stages. Importantly, mRNA expression of PbSERA1–4 could hardly be detected during the first 38 h post infection, whereas expression was strongly upregulated 50 h post infection (Fig. 1C). Further characterization of the SERA3 promoter largely confirmed the RT-PCR results. We cloned the complete 5′ UTR of SERA3 in front of GFP within a *P. berghei* transfection plasmid. Transfected *P. berghei* parasites do not express GFP in early liver stages but show a strong GFP expression towards the end of the exoerythrocytic stage, in particular during merozoite formation (Fig. S2).

Similarly to other SERA protease genes that have been identified (Arisue *et al*., 2007; McCoubrie *et al*., 2007), PbSERA genes are arranged in a tandem cluster (Fig. 1B) that contains two serine-type SERAs (PbSERA1, -2) and three cysteine-type SERAs (PbSERA3, -4 and -5). Alignment of the amino acid sequences of the five PbSERAs and PfSERA5 of *P. falciparum* revealed highly conserved areas including the protease-like domain in the central region and areas of low homology at the N- and C-termini of the proteins (Fig. S1). RT-PCR analysis was used to determine stage-specific mRNA expression of SERA genes throughout the life cycle of the parasite. Messenger RNA was isolated from two mosquito stages (oocysts and salivary gland sporozoites) and three mammalian stages (late liver schizonts, detached cells/merosomes, infected erythrocytes) and subjected to RT-PCR using primers specific for each PbSERA cDNA (Fig. 1D). Whereas PbSERA1 mRNA expression was detected in oocysts, late liver stage parasites, detached cells/merosomes and infected RBCs, PbSERA2–4 gene transcription was restricted to mammalian stages. On the other hand, PbSERA5 mRNA could only be detected in mosquito stages, confirming previous results (Aly and Matuschewski, 2005).

### PbSERA3 is processed in late liver stages

**Fig. 2:**
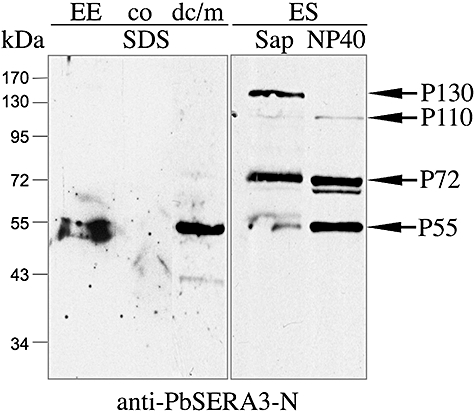
Processing of PbSERA3 in liver and blood stages. Saponin and NP40 extracts of erythrocytic stage (ES) were made and compared with whole-cell SDS lysates of detached cells/merosomes (dc/m), FACS-sorted HepG2 cells containing 50 h exoerythrocytic schizonts (EE) cells and non-infected HepG2 cell lysates (co) by Western blot analysis using an anti-PbSERA3-N antiserum.

To produce an antiserum that specifically recognizes PbSERA3, rats were immunized with the variable N-terminal region of the protein. It is well established that SERA proteases of *P. falciparum* undergo several steps of processing but nothing was known about processing of the *P. berghei* homologues. To investigate processing of PbSERA3 during blood and liver stages, we performed Western blot analysis using the anti-PbSERA3-N antiserum (Fig. 2). Several PbSERA3 protein fragments were detected in cell extracts of blood stage parasites, infected HepG2 cells and also in detached cells and merosomes. Whereas in extracts of blood stage parasites, full-length PbSERA3 (130 kDa) and processed forms of 72 and 55 kDa were recognized by the antiserum, a processed 55 kDa PbSERA3 was the main fragment in late infected HepG2 cells and in detached cells/merosomes detected by the antiserum used for this experiment. The 55 kDa species most likely corresponds to the N-terminal fragment P47*,* which is the result of SERA5 processing in *P. falciparum* blood stage parasites (Li *et al*., 2002) and does not correspond to the mature PbSERA3 protease. Saponin treatment, which releases the content of the PV, suggested that in blood stage parasites, PbSERA3 is first secreted into the PV and subsequently processed, most likely by other proteases in the PV (Fig. 2).

### TAP-tagging of PbSERA3

**Fig. 3:**
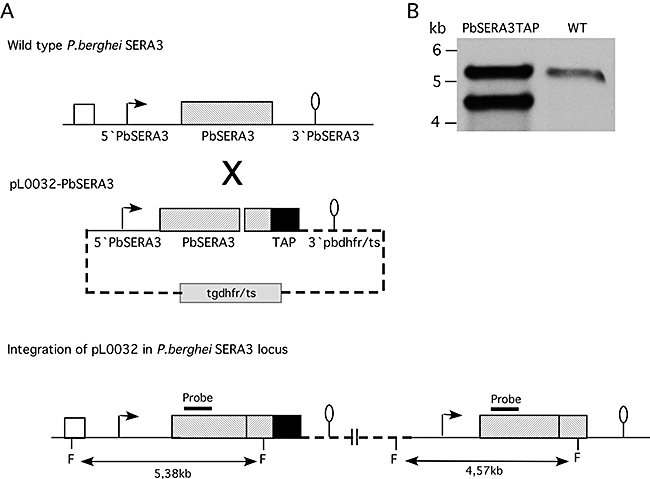
Integration of a TAP-tagged copy of PbSERA3 into the SERA locus. Shown are schematic representations detailing the outcome of the expected integration event. A.Wild-type PbSERA locus showing part of the PbSERA2 gene (white box) and the PbSERA3 gene (grey box) with 5′ UTR and 3′ UTR (upper part). The pL0032-PbSERA3 targeting construct including the 5′ UTR and full-length PbSERA3 gene (without STOP codon) fused to a TAP-tag (black box), the 3′ pbdhfr/ts sequence and a selectable tgdhfr/ts marker cassette (middle part) was integrated into the PbSERA3 locus (lower part). F = FnuI restriction sites. The expected fragments are indicated. B.Southern blot analysis showing the correct integration event. Wild-type parasites and transfected parasites were isolated from infected mice and genomic DNA was prepared. Upon FnuI digestion, Southern blot analysis was performed using a PbSERA3-specific DNA probe. The location of the probe is indicated in (A).

To further characterize processing of PbSERA3 in adherent infected HepG2 cells, we used single cell analysis using IFA techniques. The aim was to use antibodies against both ends of the protein for staining of different parasite liver stages, assuming that colocalization of the two antibodies would represent detection of the full-length protein whereas different staining patterns would indicate processing. To detect the N-terminus we used the antiserum described above and for detection of the C-terminus, we generated a transgenic parasite strain, expressing full-length PbSERA3 fused to a TAP-tag at the C-terminus (Fig. 3A). Southern blot analysis was used to confirm the integration of the tagged PbSERA3 DNA sequence into the correct gene locus (Fig. 3B). Expression of this additional tagged SERA protease had no effect on parasite development throughout the life cycle (Fig. S3).

**Fig. 4:**
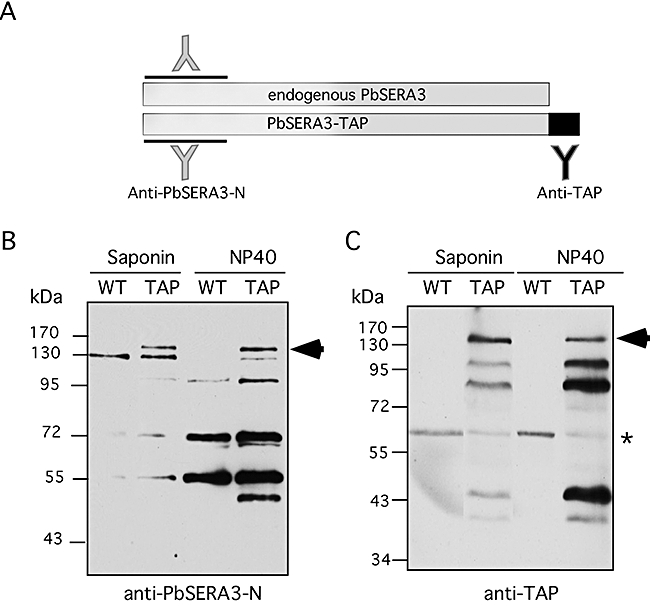
Western blot analysis of wild-type PbSERA and PbSERA3-TAP-expressing *P. berghei* blood stage protein extracts. A.Schematic representation of endogenous PbSERA3 protein and TAP-tagged PbSERA3. The epitopes against which the different antibodies are directed (anti-PbSERA3-N and anti-TAP) are indicated. B and C. Wild-type (WT) or transgenic parasites (TAP) were initially lysed with saponin to extract proteins present in the PV and subsequently treated with NP40 to extract soluble parasite proteins. Western blot analysis was performed using antibodies directed against the PbSERA3 N-terminus (B) or the TAP-Tag (C). Molecular weight markers (in kDa) are shown on the left side of each blot. The arrows indicate the full-length TAP-tagged PbSERA3 and the asterisk in C labels an unspecific band.

Protein extracts of purified wild-type (WT) *P. berghei* blood stage parasites and transgenic parasites expressing additionally to the endogenous PbSERA3, TAP-tagged PbSERA3, were first analysed by Western blotting to demonstrate a normal processing of the tagged protein. Purified WT or transgenic parasites were treated with saponin to initially extract proteins present in PV (Fig. 4B). A subsequent lysis with NP40 was used to extract soluble proteins from the parasite. As shown in a schematic illustration in Fig. 4A, human IgG is expected to detect TAP-tagged full-length and processed C-terminal fragments of PbSERA3-TAP, but no reactivity is expected for the endogenous PbSERA3. On the other hand, the anti-PbSERA3-N antiserum should recognize both full-length proteins and the N-termini of the processed forms of PbSERA3 as well as TAP-tagged PbSERA3. For both WT and transgenic parasite preparations, full-length proteins were primarily found in the PV (saponin extraction) whereas processed forms were mainly detected in parasite extracts (Fig. 4B) confirming the results presented in Fig. 2. As these fragments are present in WT and transgenic parasite and because they are also not detected by the anti-TAP human IgG, it can be concluded that they lack the C-terminus of the protein. Importantly, during the blood stage of the parasite TAP-tagged PbSERA3 appeared to be processed similarly to WT PbSERA3 with the exception of a processed form of about 50 kDa, which was not present in extracts of WT parasites. The four main protein fragments detected in saponin- and NP40-extracts of transgenic parasites by human IgG correspond to four protein species that include the C-terminus and the TAP-tag as illustrated in Fig. 4A. The simultaneous occurrence of many PbSERA3 peptides in Western blot experiments in comparison to relatively limited number of fragments in Western blot analysis of *P. falciparum* cell extracts (Li *et al*., 2002) reflects most likely the non-synchronous growth of *P. berghei* in mice. To confirm this assumption, we synchronized *P. berghei* blood stage parasites (WT parasites and PbSERA3-TAP expressing parasites) *in vitro* and repeated the Western blot analysis with lysates of highly enriched schizonts (Fig. S4). As expected, processing was more pronounced when compared with lysates of mixed cultures. The prominent 72 kDa protein detected in mixed cultures by the antiserum directed against the N-terminus is not present in lysates of synchronized cultures (Fig. S4A). However, when the blot was probed with anti-TAP antiserum (Fig. S4B), no difference was seen to the experiment with mixed culture lysates, suggesting that N-terminal but not C-terminal processing occurs in blood stage schizonts.

### Subcellular localization of PbSERA3

**Fig. 5:**
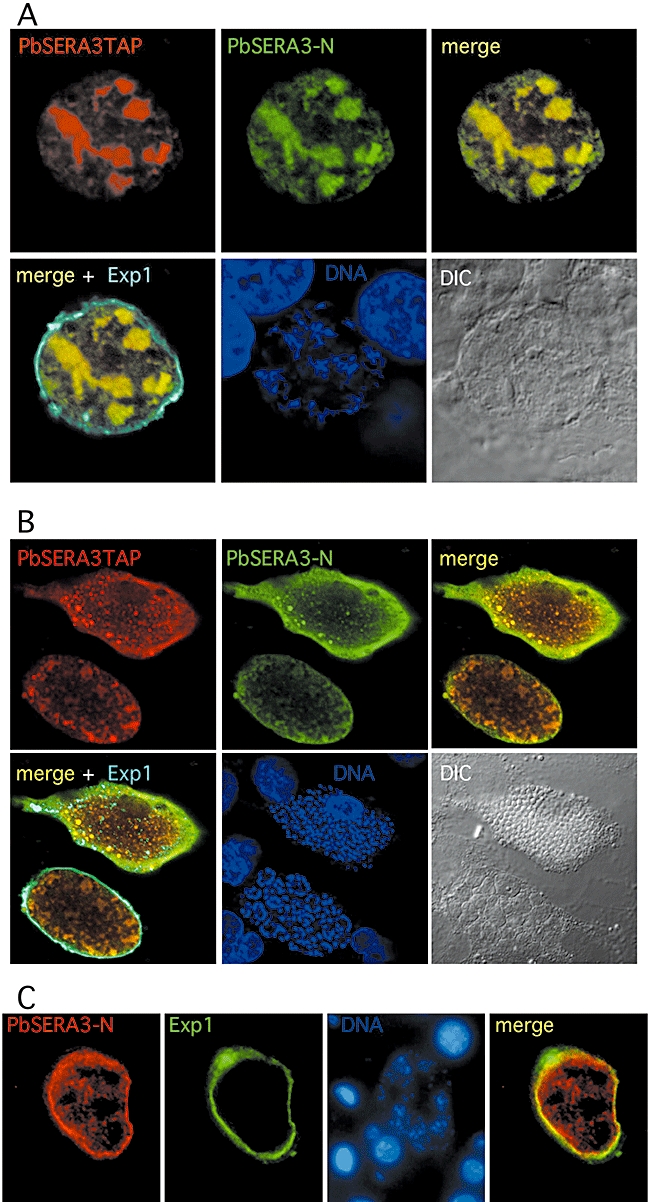
PbSERA3 localization in *P. berghei*-infected HepG2 cells and in hepatocytes. Infected HepG2 cells were fixed 48–60 h post infection and stained with anti-TAP (red), anti-PbSERA3-N (green), anti-ExpI (cyan) to visualize the PVM and the DNA dye DAPI (blue). (A) Schizont stage; (B) cytomere and merozoite stage; (C) section of a *P. berghei*-infected liver stained with anti-PbSERA3-N (red), anti-ExpI (green) and DAPI (blue). Bar = 10 μM.

Western blot analysis of blood stage parasite extracts suggested that processed PbSERA3 forms exists in the PV and in the parasite. To determine the subcellular localization of PbSERA3 in late liver stage parasites, indirect immunofluorescence analysis was performed. To prove whether the TAP-tagged and the endogenous versions of PbSERA3 colocalize, we stained schizont-infected HepG2 cells with both human IgG to detect TAP-tagged PbSERA3 and with the antiserum directed against the N-terminus of PbSERA3 (Fig. 5A). The vast majority of the detected proteins indeed colocalized in defined regions of the parasite, indicating that the TAP-tag does not alter the localization of the tagged protein. Using the PbSERA3-N antiserum a similar staining pattern was also seen in HepG2 cells infected with WT parasites (Fig. S5), confirming that additional expression of TAP-tagged PbSERA3 has no effect on the localization of the protein. From this experiment, it can be concluded that human IgG is indeed suitable for detection of the TAP-tagged C-terminus of PbSERA3 at the correct location. Importantly, in fixed liver sections of *P. berghei*-infected mice, the staining pattern obtained by anti-PbSERA3-N antiserum detection resembled the *in vitro* acquired results with PbSERA3-N localizing mainly in the parasite cytoplasm and close to the PVM (Fig. 5B and C).

**Fig. 6:**
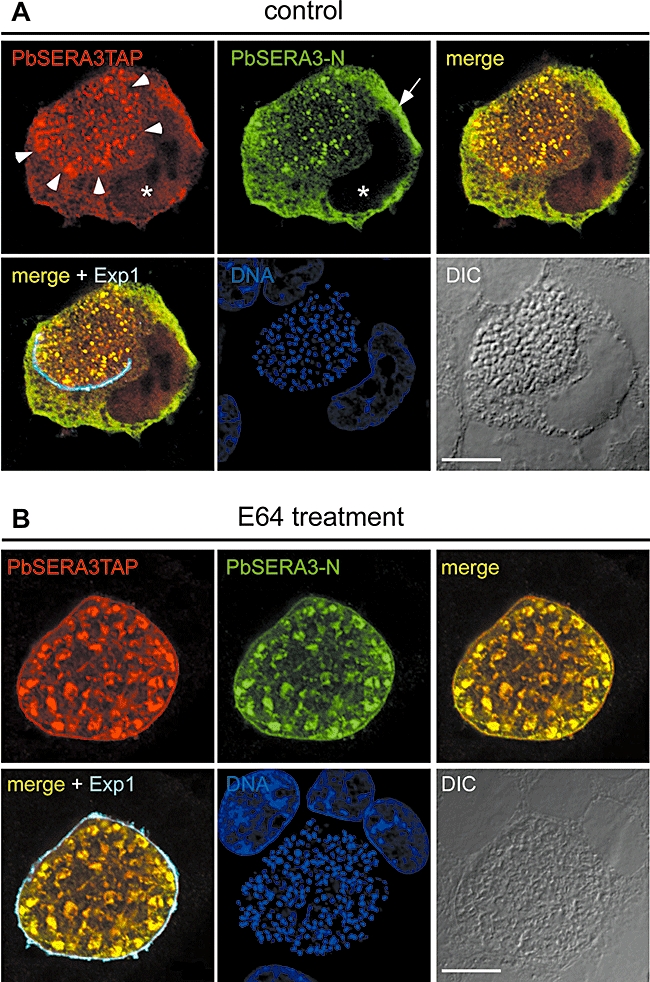
Inhibition of cysteine protease activity by E64 inhibits PbSERA3 release in the host cell cytoplasm. A total of 48 hpi infected HepG2 cells were treated with E64 (B) or were left untreated as a control (A) and cultured for additional 15 h. Cells were fixed and stained as described above. The asterisk in (A) labels the host cell nucleus, which contains already the TAP-tagged C-terminus of PbSERA3 but not its N-terminus. Arrowheads mark the parasite dimensions in the infected cell. The arrow indicates an area of high PbSERA3-N concentration. Note that the concentration of TAP-tagged C-terminus is clearly lower (red staining). Bar = 10 μM.

In order to inhibit SERA processing, we treated infected HepG2 cultures 48 hpi with E64 and stained the cells with anti-TAP and anti-PbSERA3-N 56 hpi. Additionally, the PVM was visualized using an anti-Exp1 antiserum. At this time point, in a substantial number of infected non-treated control cells, PbSERA3 was found in the host cell cytoplasm whereas in E64-treated cells, PbSERA3 was restricted to the parasite. In Fig. 6, representative images are depicted. When merozoites have been formed, the PVM ruptures, releasing PbSERA3-N and C into the host cell cytoplasm (Fig. 6A). However, a closer examination of the staining patterns revealed some interesting differences in the distribution of PbSER3-N and C fragments. PbSERA3-N was found equally distributed over the remaining parasite structure and the host cell but was absent from the host cell nucleus. In contrast, the TAP-tagged C-terminus of PbSERA3 was clearly more concentrated to the parasite. Staining of the host cell cytoplasm with anti-TAP was less pronounced than was seen for anti-PbSERA3-N staining. Despite the reduced occurrence of PbSERA3-C in the host cell, it was found in the nucleus of the host cell. Together, the differences in the staining pattern clearly confirm PbSERA3 processing at this stage in non-treated control cells. On the other hand, the PVM in E64-treated cells appeared to be intact and in the vast majority of infected cells, no PbSERA3 translocation to the host cell cytoplasm was observed (Fig. 6B). Importantly, PbSERA3-N and PbSERA3-TAP colocalized almost completly, indicating that PbSERA3 is indeed inhibited by E64 treatment.

**Fig. 7:**
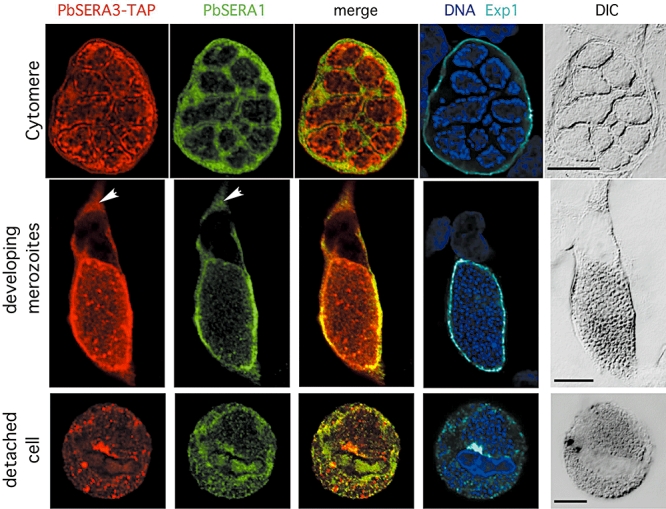
PbSERA3-C and PbSERA1-M localization in infected HepG2 cells. *P. berghei*-infected HepG2 cells were fixed at different time points post infection and stained with anti-TAP (red), anti-PbSERA1-M mAb (green), anti-Exp1 (cyan) and DAPI (blue). Arrows indicate SERA localization in the host cell cytoplasm. For easier interpretation, only images obtained with anti-TAP and anti-PbSERA1 were merged. Yellow colour indicates colocalization. Bar = 10 μM.

With the transgenic parasite line expressing a TAP-tagged PbSERA3 we now have a valuable tool to perform colocalization experiments with other PbSERAs. Together with a panel of specific antibodies, this should help us to analyse PbSERA expression, localization and processing during the liver stage of the parasite in detail. Figure 7 provides the first hint that PbSERA localization might vary considerably during the exoerythrocytic parasite stage. Quadruple labelling experiments using human IgG, mouse anti-PbSERA1, chicken anti-Exp1 and DAPI were used to determine SERA localization at different parasite stages. In cytomere-stage parasites, PbSERA3 localized mainly in the parasite cytoplasm and the PVM, with little protein located in the PV (Fig. 7, upper panel). Interestingly, at this stage, PbSERA1 was predominantly detected in the PV, suggesting a different localization of both PbSERA proteins. When merozoites have been formed, both antibodies label the already damaged PVM and the host cell cytoplasm (Fig. 7, middle panel). Finally, in detached cells, the PVM is completely disintegrated and PbSERA-1 and -3 are found in the host cell in distinct foci, which do not completely overlap (Fig. 7, lower panel). It should be noted that in cell extracts of detached cells/merosomes, only processed PbSERA3 protein was detected by Western blotting (Fig. 2), suggesting that TAP detection at this stage visualizes the processed C-terminus of the protein.

## Discussion

Here, we present for the first time evidence that four putative proteases of the PbSERA family are expressed in late liver stages and that PbSERA3 becomes processed during merozoite development. In the activated state, PbSERA3 might contribute to the observed PVM destruction and merozoite liberation as has been postulated for SERA5 during the blood stage of *P. falciparum* (Hodder *et al*., 2003). Although there is no doubt that cysteine proteases mediate PVM destruction and facilitate merozoite release from hepatocytes, additional factors might be required to guarantee release of merozoites from merosomes. It has recently been shown that merosomes migrate out of the liver and reach the lung (Baer *et al*., 2007b). It might well be that in the microvasculature, a pressure-driven merozoite release occurs similarly to what has been described for *P. falciparum* merozoite release from erythrocytes (Glushakova *et al*., 2005). Our own *in vitro* live imaging of *P. berghei*-infected hepatocytes supports this view. We observed that merozoites are not all released at once from merosomes but are liberated in several convulsive eruptions over a period of about 1 h. After the release of 10–20 merozoites the merosome membrane is closed again until the next eruption (V.T. Heussler, unpubl. obs.). This form of merozoite release might well be triggered by increased osmotic pressure, which decreases upon merozoite release and again increases once the membrane is re-sealed. However, the fact that the entire process of PVM degradation and merozoite liberation can be blocked by different protease inhibitors demonstrates clearly that proteases appear to act as initiators of merozoite release.

We found that mRNA expression of four PbSERA proteases was strongly upregulated briefly before merozoite development. Furthermore, we could show by IFA that, at this stage, PbSERA1 translocated predominantly into the PV. Upon disruption of the PVM, PbSERA1 and PbSERA3-C were localized in the host cell cytoplasm, suggesting a role in the subsequent host cell death. SERA proteases have also been proposed to mediate parasite liberation from *P. falciparum*-infected erythrocytes with PfSERA5 being processed briefly before merozoite liberation (Hodder *et al*., 2003) and it appears likely that the molecular mechanisms of *Plasmodium* merozoite liberation from infected erythrocytes and hepatocytes are similar. It has already been shown that *P. falciparum* SERA5 has a weak proteolytic activity (Hodder *et al*., 2003), but the physiological roles of the different SERAs and their substrates in infected cells are still not known (McCoubrie *et al*., 2007). Approaches to knockout the different *P. falciparum* SERAs expressed during the blood stage either did not generate an apparent phenotype or were lethal (Miller *et al*., 2002; McCoubrie *et al*., 2007). Obviously, knockout of some PfSERAs can be compensated by upregulating the expression of others (McCoubrie *et al*., 2007) and thus alternative approaches like inducible expression or stage-specific expression (Carvalho *et al*., 2004) have to be considered in the future to determine the function of the individual SERAs in blood and liver stages. As we show here that PbSERA proteases localize to different compartments in infected hepatocytes, it is also possible that not only expression levels but also the change in protein localization might compensate for the lack of certain SERAs in knockout parasites. Staining of large liver stage parasites is much easier to perform and to interpret than staining of infected RBCs and thus *P. berghei*-infected hepatocytes might turn out to be a valuable tool to analyse the localization and the function of SERA proteases in general. The most important evidence that SERA proteases are involved in parasite liberation comes indeed from knockout studies performed in *P. berghei* (Aly and Matuschewski, 2005). These authors had demonstrated that PbSERA5 expression is restricted to the insect stage and thus selection of knockout parasite strains during the blood stage was possible. Parasites lacking functional PbSERA5 did not show defects in mosquito infection or in the development of sporozoites within oocysts. However, PbSERA5-knockout sporozoites were not able to leave mature oocysts and no salivary gland infection was observed. Another intriguing question is why *Plasmodium* parasites express several rather similar proteases at the same time. Our observation that the localization of these proteases differs considerably within infected cells provides an explanation for this simultaneous expression. Alternatively, the different SERA proteases might be arranged in cascades with one SERA processing another member of the family similarly to what is known for caspases in metazoans. As we expect that mature PbSERA proteases, once translocated to the cytoplasm of the infected hepatocyte, are responsible for executing host cell death, this would indeed be a striking analogy with initiator caspases and executor caspases triggering apoptosis in other organisms. Interestingly, the genome of *Theileria annulata*, which is a closely related parasite to *Plasmodium*, contains also a SERA gene (McCoubrie *et al*., 2007). As *Theileria* parasites actively inhibit host cell apoptosis during the schizont stage but kill their host cell during merozoite release (Heussler *et al*., 2002), it will be highly interesting to determine the expression and the function of the putative protease during parasite liberation from infected leukocytes. For *Plasmodium*-SERAs, the next challenge is to decipher their function in detail, in particular to define their role in the execution of host cell death. The work presented here provides a good basis for further analysis of SERA function, and some exciting findings can be expected in the near future.

## Experimental procedures

### Experimental animals

Animals were from Charles River Laboratories. All animal work was conducted in accordance with European regulations and approved by local state authorities.

### PbSERA gene prediction and amino acid sequence alignment

Homology searches for *P. berghei* sequences similar to PySERA1–5 and the annotated PfSERA1–9 genes were performed with the basic local alignment search tool (blast) program of the GeneDB website or of PlasmoDB website. cDNA sequences were translated and then aligned using the CLUSTAL W programme of MacVector.

### Transcript detection

For RT-PCR analysis, total RNA was isolated from 6 × 10^5^ WT salivary gland sporozoites, 10^6^ WT oocyst sporozoites, infected HepG2 cells at different time points post infection, detached cells/merosomes or from 0.15% saponin-treated (Sigma) blood stage parasites (RBC stage) using the RNA Extract Kit II (Machery and Nagel). First strand cDNA was synthesized with the Superscript^TM^ First-Strand Synthesis System for RT-PCR (Invitrogen) using 200 ng of each total RNA. Target cDNAs were amplified using the following primer sets: PbSERA1/for (5′-TTAGATGCAGCCGACACAAG-3′) and PbSERA1/rev (5′-TACTCCATTTCGCAGCACAA-3′); PbSERA2/for (5′-TTCCCTTTCACCACAACCTC-3′) and PbSERA2/rev (5′-TCACATTTGTTCGTTTCTGGA-3′); PbSERA3/for (5′-ATGGCACGTCTCTCATCAAT-3′) and PbSERA3/rev (5′-TGTGGTGAAAATTGAACTCTGAA-3′); PbSERA4/for (5′-CACGAAATTAATACGCAAACCT-3′) and PbSERA4/rev (5′-TCATTAGTGTGTGTTTCCCATT); PbSERA5/for (5′-TCTGGAACAAGCAATTTACAAAAA-3′) and PbSERA5/rev (5′-TCAGCGAATCCAAGTCCTTT-3′). As an internal control, a primer set (Pbtubulin/for (5′-TGGAGCAGGAAATAACTGGG-3′) and Pbtubulin/rev (5′-ACCTGACATAGCGGCTGAAA-3′) that annealed to *P. berghei* tubulin was used. All RNA preparations were free of genomic DNA (gDNA) contamination as no PCR product was obtained when reverse transcriptase has been omitted from the RT-PCR (negative control).

### *In vitro* infection of HepG2 cells

Human hepatoma cells (HepG2) were obtained from the European cell culture collection. Cells were cultivated at 37°C and 5% CO_2_ in EMEM (Gibco), containing 10% fetal calf serum, 1% l-glutamine, 1% penicillin/streptomycin and 1% MEM non-essential amino acids (all PAA Laboratories GmbH, Pasching, Austria). The cells were passaged by trypsinization every 4–6 days and for infection, 10^5^ cells were seeded per coverslip in a 24-well plate. *P. berghei* WT (ANKA) or transgenic sporozoites were prepared from dissected salivary glands of infected *Anopheles stephensi* mosquitoes and incubated with HepG2 cells for 2 h. After washing, the infected cultures were incubated at 37°C until the indicated times.

### SDS-PAGE and Western blotting

Parasite proteins obtained from 55% Nycodenz-enriched blood stage schizont preparation or collected floating cells/merosomes were separated on 12% SDS-PAGE reducing gels and transferred to nitrocellulose membranes. Membranes were probed with rat antisera directed against the N-terminus of PbSERA3 (PbSERA3-N) or human gamma-globulins (anti-TAP, Sigma). Horseradish peroxidase-conjugated goat anti-rat or anti-human IgG (Pierce) were used for detection, and bands were visualized using enhanced chemiluminescence Pico Detection Kit (Pierce).

### Immunofluorescence microscopy

For analysis of SERA localization in late liver stages, infected HepG2 cells were fixed with 4% formaldehyde, permeabilized with ice-cold methanol and incubated with primary antibodies (1:400 in 10% FCS diluted in PBS) or human gamma-globulin (10 μg ml^−1^ in 10% FCS diluted in PBS). Bound antibodies were detected using anti-human Alexa Fluor 594-, anti-rat Alexa Fluor 488- or anti-chicken Cy5-conjugated secondary antibody (Molecular Probes, Leiden, the Netherlands). To identify the PVM of intracellular parasites, cells were stained with a parasite-specific chicken anti-PbExp1 antibody. Nuclei of the cells were visualized with a 10 μg ml^−1^ aqueous solution of DAPI (Sigma-Aldrich, Germany). Immunofluorescence-labelled cells were examined by confocal microscopy using the Olympus FV1000 (SIM-scanner and spectral detection).

### Immunohistological staining

For analysis of PbSERA3 localization *in vivo*, female C57BL/6 mice were infected intravenously with 2.5 × 10^5^*P. berghei* WT (ANKA) sporozoites. Forty-eight hours post infection, mouse liver samples were fixed in HOPE Solution I (Hepes Glutamic Acid buffer mediated organic Solvent Protection effect; DCS, Hamburg) and were embedded in paraffin. Sections of 2 μm were treated with 0.5% Triton X-100 diluted in PBS and stained with rat anti-PbSERA3-N and chicken anti-Exp1 antibodies, diluted to 1:100, followed by detection with anti-rat-Alexa 594 and antichicken-Cy2. DAPI was used to stain the nuclei of the host cell and the parasite.

### Antibodies

#### Expression and purification of recombinant GST fusion proteins

RT-PCR was used to clone PbSERA1, and PbSERA3 cDNA. BamHI/EcoRI sites and XhoI sites were introduced at the end of forward and reverse primers respectively (restriction enzyme sites are underlined): SERA3/for (5′-GTGGATCCATGGCACGTCTCTCATCAAT-3′) and SERA3/rev (5′-GTCTCGAGATGTGGTGAAAATTGAACTCTGAA-3′); SERA1/for (5′-GTGTGAATTCTTAGATGCAGCCGACACAAG-3′) and SERA1/rev (5′-GACTCGAGTTATCCTTCTCCAGTTGGTTGATG-3′). The resulting PCR products were ligated into the appropriate pGEX vectors and expressed in *Escherichia coli* BL21 cells (Stratagene) as glutathione *S*-transferase (GST) fusion proteins. GST fusion proteins of PbSERA1 (Leu^877^-Gly^910^) or PbSERA3 (Met^1^-Phe^235^) were harvested by suspending bacteria expressing SERA proteins in 10 ml buffer A (10 mM EDTA in PBS) in the presence of Complete™ Protease Inhibitor mixture tablets (Roche Molecular Biochemicals) for 30 min followed by sonification. The recombinant SERA fusion proteins were purified from the supernatant using glutathione-sepharose as described by the manufacturer (Amersham Biosciences). Twenty micrograms of each purified fusion protein was mixed with Freund's complete adjuvant and used to immunize rats (Lewis, female 3 months old), followed by multiple boosting.

#### Hybridoma production

Folowing immunization, blood samples were collected to test serum reactivity against the SERA1-C protein. From a positive mouse lymph node, cells were isolated and fused to the mouse myeloma cell line X63Ag8.653. Supernatants were screened by direct ELISA and single-cell clones were isolated by limited dilution.

#### Purification of antibodies

To collect polyclonal rat serum, immunized animals were killed and heperanized blood was collected by cardiac puncture. Serum was obtained after centrifugation. Monoclonal antibodies were isolated by applying the cell culture supernatant to a Protein G-Sepharose column (Amersham Biosciences). Total IgG was eluted from the column and dialysed against PBS.

### pL0032-SERA3 plasmid design and transfection

DNA was amplified by PCR from *P. berghei* blood stage parasite gDNA template using the Phusion Taq DNA polymerase High Fidelity enzyme (Finnzyme). Preparation of gDNA was performed using the Blood DNA Extract Kit (Qiagen). The *P. berghei* TAP transfection plasmid pL0032, for homologous recombination into the genome, was obtained from MR4. The 5′ UTR and the complete PbSERA3 ORF targeting sequence were obtained by amplification of *P. berghei* gDNA using the primer set: SERA3-TAP/for (5′-GCTCTAGATTTAACAATAAACTTTGCAAAATAGTGAAT-3′) and SERA3-TAP/rev (5′-CATGCCATGGACATAACAGAAGAGACATTTGTTTTTTCC-3′). The resulting fragment was cloned into the NcoI/XbaI cloning sites of pL0032 in frame with the TAP-tag coding sequence. The plasmid was linearized for transfection using the unique restriction site XcmI. Schizont-stage parasites were transfected with 5 μg of purified plasmid DNA (Machery and Nagel Kit PC100), as previously described (Janse *et al*., 2006).

### Southern blot

For isolation of parasite gDNA, infected mice were exsanguinated at 3–5% parasitemia and gDNA was isolated from whole blood as described above. Manipulation of recombinant DNA and analysis of nucleic acids by Southern blot hybridization were carried out using a non-radioactive labelling kit according to manufacturers protocol (Amersham Biosciences).

### Comparison of wild-type versus transgenic SERA3-TAP parasite development

For counting oocysts, midguts of WT or SERA3-TAP transgenic *P. berghei*-infected *A. stephensi* mosquitoes were stained in 0.5% mercurochrome in 0.9% NaCl 10 days after a blood meal.

Monitoring parasitemia in mice: sporozoites were prepared from salivary glands of *A. stephensi* mosquitoes 21 days after an infectious blood meal. A total of 30 000 WT or SERA3-TAP transgenic *P. berghei* sporozoites were injected intravenously into three mice for each parasite strain. Parasitemia was monitored by blood smears.

### FACS sorting of exoerythrocytic GFP-expressing *P. berghei* parasites

HepG2 cells were seeded 24 h prior infection in 24-well plates. GFP-expressing *P. berghei* sporozoites from five to 10 well-infected mosquitoes were used to infect each of three wells. Fifty hours post infection, cells were detached by Accutase treatment, pooled in 10 ml media and subsequently FACS sorted. Sorted infected cells were immediately lysed and used for Western blot analysis as described above.

## Supplementary material

The following supplementary material is available for this article online:

Fig. S1 Comparison of PbSERA1–5 and *P. falciparum* SERA5. Multiple alignment of amino acid sequence of *P. berghei* SERAs with PfSERA5. Gaps are introduced to maximize homology. Light grey shading indicates similarities. Dark grey shadings indicate identities. The PfSERA5 processing sites by PfSUB1 (Yeoh *et al*., 2007) are indicated by black shading and white letters. Asterisks label the putative catalytical triade of SERA proteases.

Fig. S2 The SERA3 5′ non-transcribed region induces late GFP expression in transgenic liver stage parasites. The entire 5′ UTR of PbSERA3 gene was cloned in front of GFP into the plasmid pL0031. *P. berghei* parasites were transfected with resulting plasmid construct. Transgenic parasites were passaged through mosquitoes and corresponding sporozoites were used to infect HepG2 cells. Live imaging was performed at indicated time points. As a control, transgenic parasites expressing GFP under the constitutive pbeef1aa promoter were used to infect HepG2 cells. Bar = 10 μM.

Fig. S3 Normal development of transgenic *P. berghei* expressing TAP-tagged SERA3. A. Insect stage. Mosquitoes were infected with wild-type (WT) or transgenic *P. berghei* parasites expressing TAP-tagged SERA3. 10 days post infection, midguts of 6 (for WT) and 8 (for transgenic parasites) mosquitoes were disected and the midguts were stained with mercurochrome before oocystes were counted. The numbers of oocystes of all individual mosquitoes are depicted on the graph. B. Mammalian stage. Sporozoites were prepared from salivary glands of infected mosquitoes and intravenously injected into mice. Blood stage parasitemia was followed for 10 days. The course of parasitemia for all individual mice is shown.

Fig. S4 WB analysis of synchronized *P. berghei* blood stages. A mixed *P. berghei* culture was synchronized as described previously (Janse *et al*., 2006). Saponin and NP40 lysates were prepared and subjected to SDS-PAGE. Upon transfer to nitrocellulose membranes, Western blot analysis using anti-PbSERA3-N (A) and anti-TAP (B) antiserum was performed.

Fig. S5 SERA3 localization in *P. berghei*-infected HepG2 cells. Infected cells were fixed 48 hpi and stained with an anti- SERA3-N antiserum (red) and with an anti-Exp1 antiserum (green). DNA was stained with Dapi.

This material is available as part of the online article from: http://www.blackwell-synergy.com/doi/abs/10.1111/

j.1462-5822.2008.01162.x
