# Supplementary figures and images for: Expression and processing of Plasmodium berghei SERA3 during liver stages
Source: Cell Microbiol. 2008 Aug;10(8):1723–34. doi: 10.1111/j.1462-5822.2008.01162.x (PMC2613260; doi:10.1111/j.1462-5822.2008.01162.x)

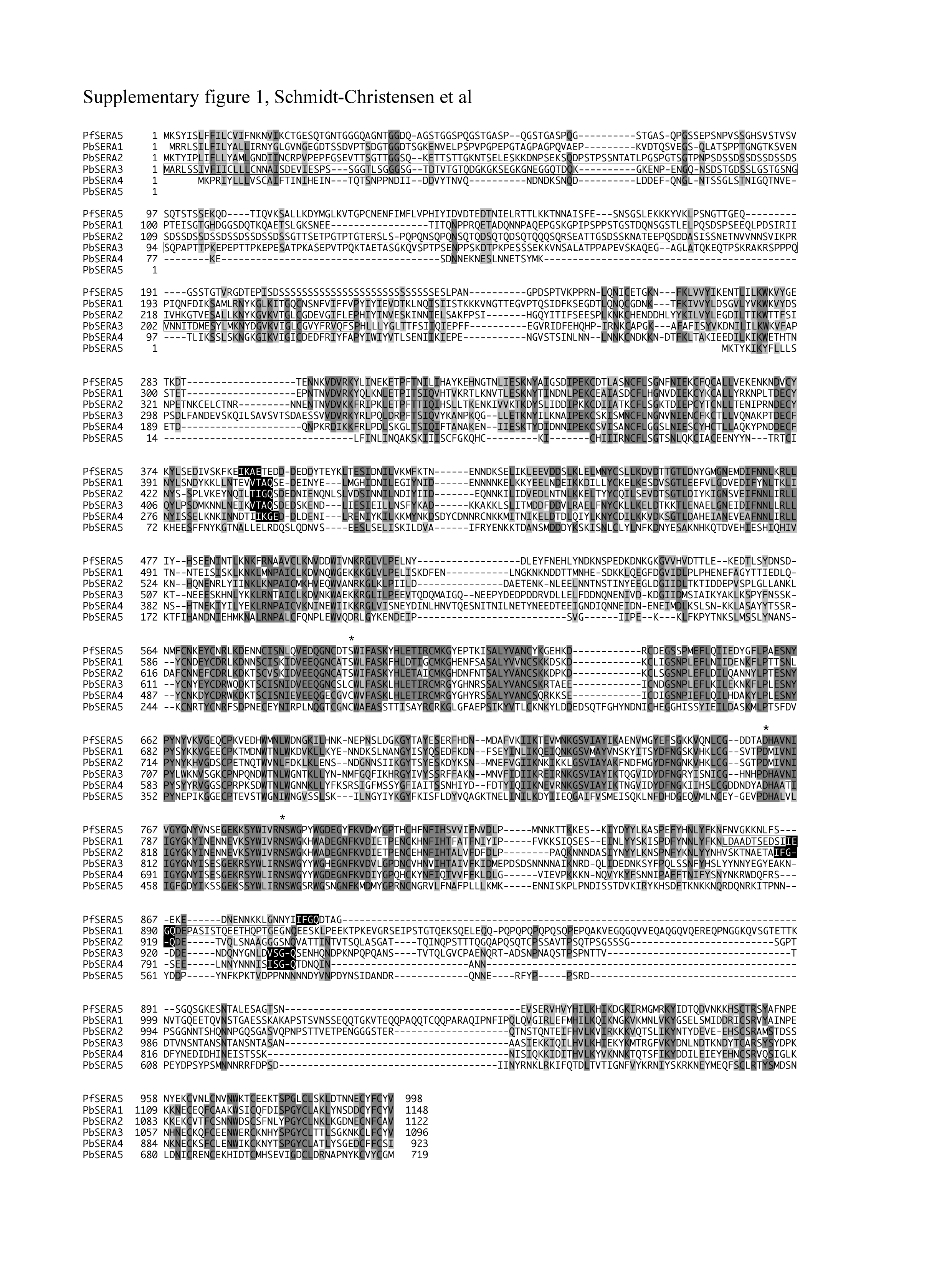

Supplement: Fig. S1 — Comparison of PbSERA1–5 and P. falciparum SERA5. Multiple alignment of amino acid sequence of P. berghei SERAs with PfSERA5. Gaps are introduced to maximize homology. Light grey shading indicates similarities. Dark grey shadings indicate identities. The PfSERA5 processing sites by PfSUB1 (Yeoh et al., 2007) are indicated by black shading and white letters. Asterisks label the putative catalytical triade of SERA proteases. [file cmi0010-1723-sd1.tif]

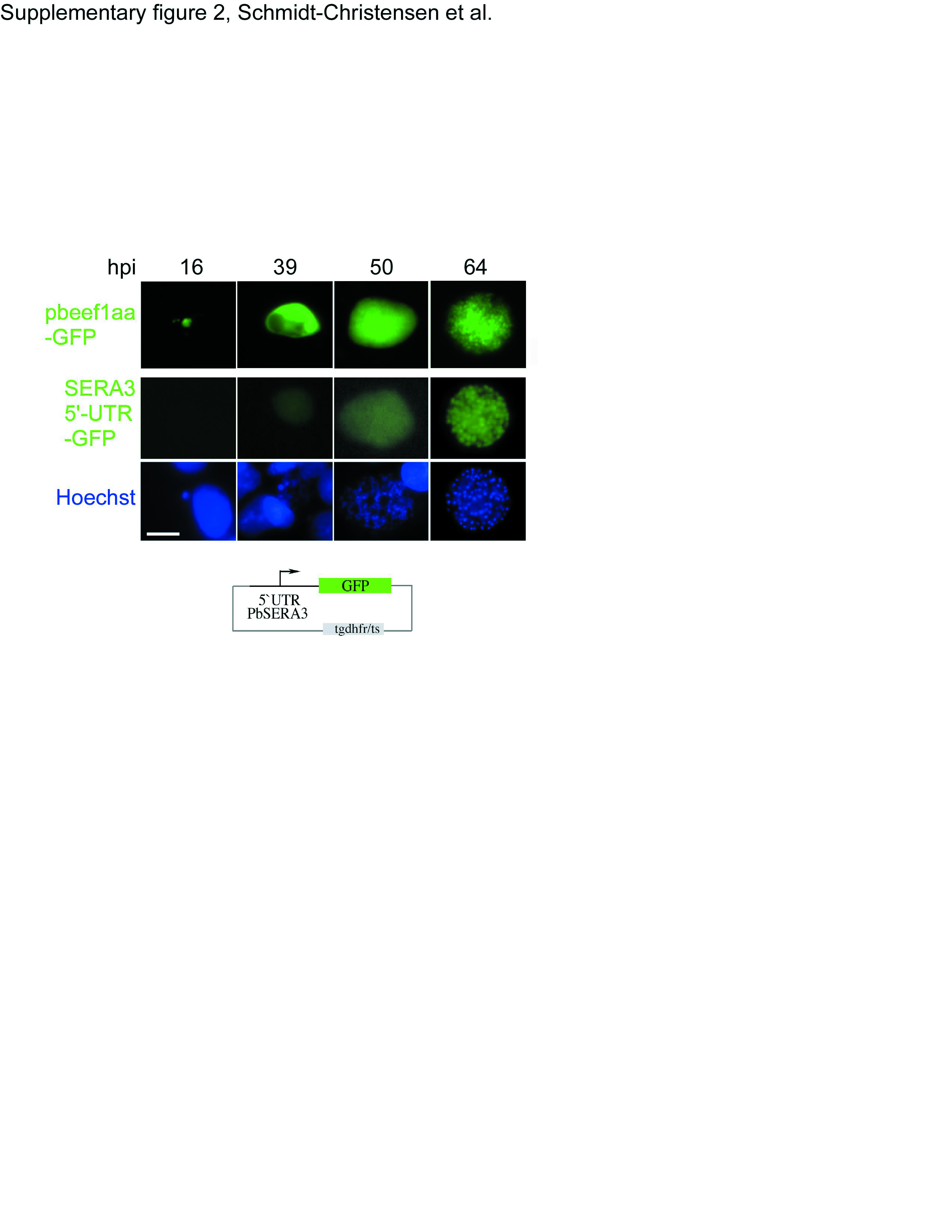

Supplement: Fig. S2 — The SERA3 5′ non-transcribed region induces late GFP expression in transgenic liver stage parasites. The entire 5′ UTR of PbSERA3 gene was cloned in front of GFP into the plasmid pL0031. P. berghei parasites were transfected with resulting plasmid construct. Transgenic parasites were passaged through mosquitoes and corresponding sporozoites were used to infect HepG2 cells. Live imaging was performed at indicated time points. As a control, transgenic parasites expressing GFP under the constitutive pbeef1aa promoter were used to infect HepG2 cells. Bar = 10 μM. [file cmi0010-1723-sd2.tif]

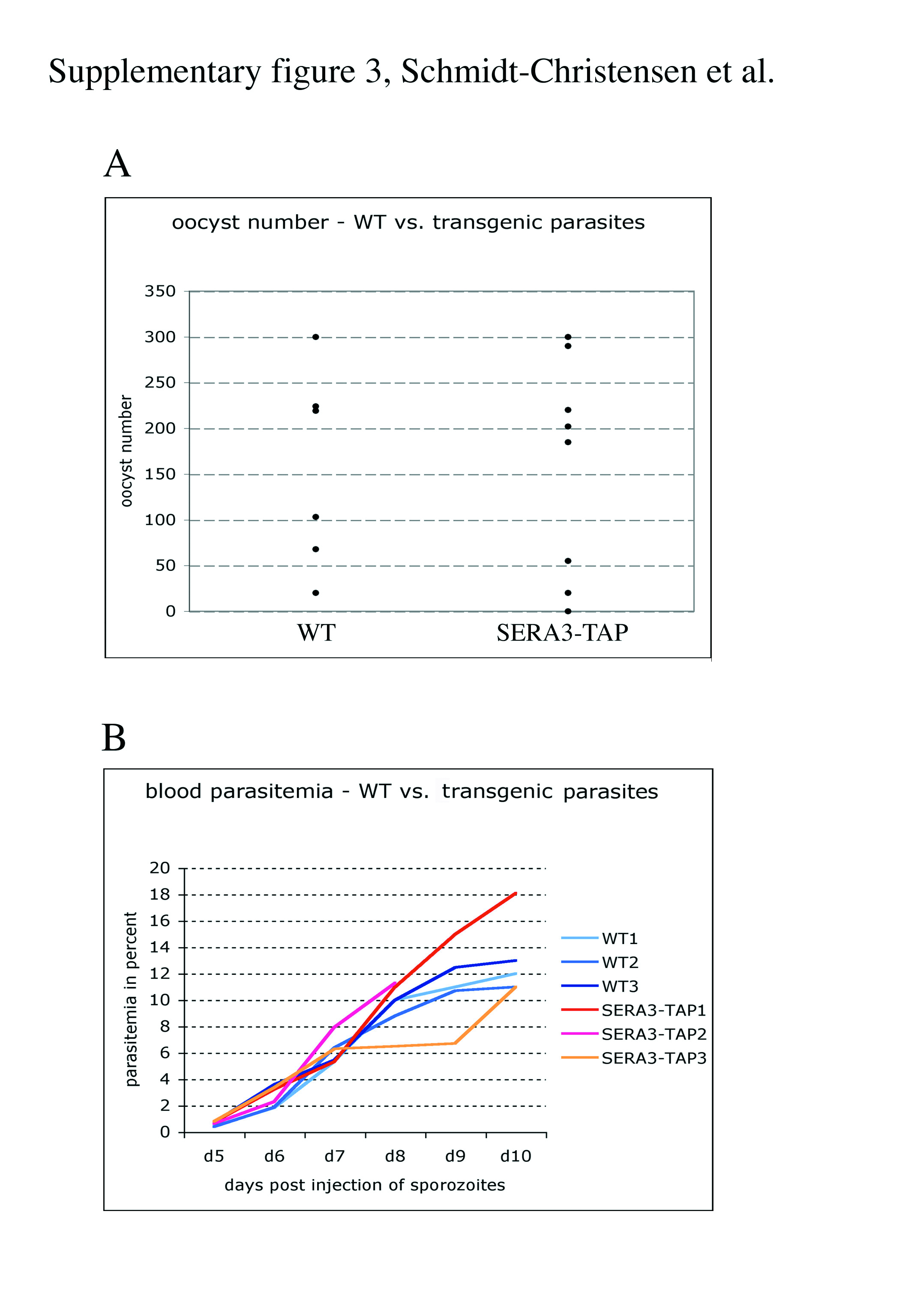

Supplement: Fig. S3 — Normal development of transgenic P. berghei expressing TAP-tagged SERA3. A. Insect stage. Mosquitoes were infected with wild-type (WT) or transgenic P. berghei parasites expressing TAP-tagged SERA3. 10 days post infection, midguts of 6 (for WT) and 8 (for transgenic parasites) mosquitoes were disected and the midguts were stained with mercurochrome before oocystes were counted. The numbers of oocystes of all individual mosquitoes are depicted on the graph. B. Mammalian stage. Sporozoites were prepared from salivary glands of infected mosquitoes and intravenously injected into mice. Blood stage parasitemia was followed for 10 days. The course of parasitemia for all individual mice is shown. [file cmi0010-1723-sd3.tif]

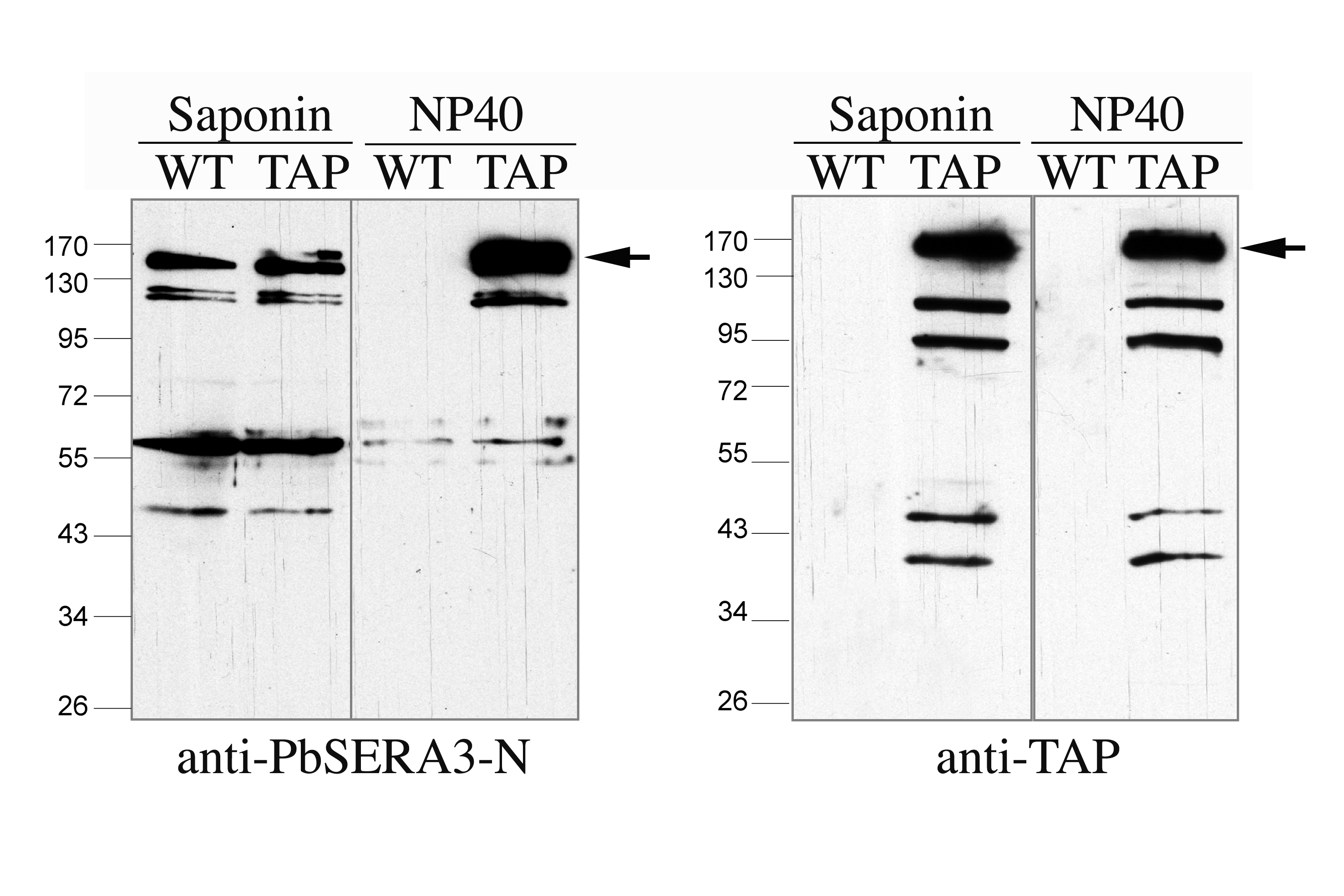

Supplement: Fig. S4 — WB analysis of synchronized P. berghei blood stages. A mixed P. berghei culture was synchronized as described previously (Janse et al., 2006). Saponin and NP40 lysates were prepared and subjected to SDS-PAGE. Upon transfer to nitrocellulose membranes, Western blot analysis using anti-PbSERA3-N (A) and anti-TAP (B) antiserum was performed. [file cmi0010-1723-sd4.tif]

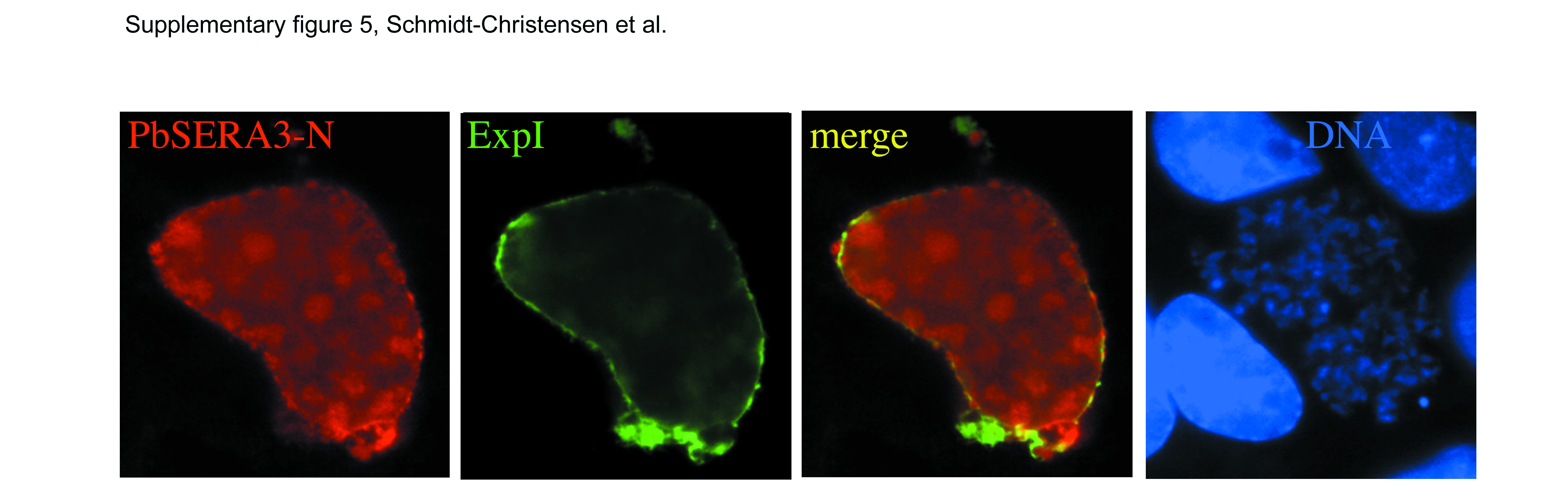

Supplement: Fig. S5 — SERA3 localization in P. berghei-infected HepG2 cells. Infected cells were fixed 48 hpi and stained with an anti- SERA3-N antiserum (red) and with an anti-Exp1 antiserum (green). DNA was stained with Dapi. [file cmi0010-1723-sd5.tif]
